# The Time Element in Therapeutics

**Published:** 1899-05

**Authors:** 


THE TIME ELEMENT IN THERAPEUTICS. A very important feature in the proper application of remedies has been overlooked by many authors and is seldom much considered by practitioners. We refer to the time which elapses between the administration of a remedy and the beginning of its action, also the duration of such effects. In the relief of many conditions, and especially in the use of combined drugs, this point is a very important consideration. In the use of digitalis it is seldom taken into consideration that it does not reach the height of its action for from four to six hours after administration, and that effect is often prolonged for a longer period. The administration of this drug, then, every three hours would hardly be justified. In several instances where it is the custom to prescribe remedies which have to a certain extent neutralizing or antagonistic effects, it is important to know these facts, otherwise the desired result may not be accomplished. For instance, it is quite a common practice to combine with digitalis remedies which counteract its effects upon the arterioles, as does nitroglycerin; but how often is the difference in the time of their action considered?

Digitalis acts very slowly, and its action is prolonged, while nitroglycerin shows its action in a few seconds, and this passes off usually in two or three hours. These drugs are frequently prescribed in one mixture, often to be given every four hours. In such a course it will readily be seen that at the latter part of the interval the patient has the acquired effects of two doses of digitalis, while the effect of the nitroglycerin has passed off. In cases where an immediate stimulation is indicated it is important to know how long we must wait for the desired effects. Nitroglycerin is immediate, handy by the stomach, or hypodermatically is rapidly diffused, and is more rapid than strychnine or many of the other commonly used stimulants. Digitalis requires some time for the development of its stimulant action, even when given hypodermatically.

Few of the works now published upon materia medica and therapeutics give the effects of different drugs. Many preparations manufactured by the pharmacist, both in the form of elixirs and tablets, show a lack of consideration of this very essential feature of scientific therapeutics. A general review of the remedies now in use, with special reference to time as an element in the physiological action, would fill an important place in the armamentarium of the practitioner.—*Kansas Medical Journal*, 1898.

